# Supplementary material for: Avian influenza A virus susceptibility, infection, transmission, and antibody kinetics in European starlings
Source: PLoS Pathog. 2021 Aug 30;17(8):e1009879. doi: 10.1371/journal.ppat.1009879 (PMC8432794; doi:10.1371/journal.ppat.1009879)
Supplement: Attachment — Submitted filename: Shriner et al 08Jul2021 PLoS Pathogens_R1 Rebuttal.docx [file ppat.1009879.s007.docx]

July 17, 2021

Dear Editors,

We are very thankful for the opportunity to revise our manuscript, Avian influenza A virus susceptibility, infection, transmission, and antibody kinetics in European starlings for further consideration in PLoS Pathogens. We are especially grateful for the accommodation made in granting us additional time to address reviewer comments.

We have responded to each of the reviewer comments, either by editing the manuscript or offering rebuttal comments. We greatly appreciate the care the reviewers to provide comments that have significantly improved the manuscript.

Our responses our detailed below in **bold**, below each reviewer comment.

Sincerely, Susan Shriner

Part I - Summary

**Reviewer #1**: The work by Ellis et al. describes the experimental infection, interspecies (mallard to starling through shared water) and intraspecies (contact transmission between starlings) experiments in order to gain more information about the possibility of starlings to act as bridge hosts between reservoir species (wild waterfowl) and spill-over hosts (poultry). Although there is still limited information about the role of passerine birds in influenza virus ecology, I think that the present study does not bring sufficient added novel information compared to the experimental infection studies cited and also nicely reviewed recently by the authors (reference 20) to justify a publication in Plos Pathogens.

**--We believe our study is a significant contribution because of the rigor and comprehensive evaluation of the potential role of starlings. The study evaluates basic infection dynamics, contact transmission, environmental/interspecies transmission, and antibody kinetics. Both the information on interspecies transmission and antibody kinetics is novel. Further, the sample sizes, fine-grained sampling, and replication are relatively unique in the field.**

In previous studies, the infection dynamics, antibody profile, and contact intraspecies transmission have already been described in starlings inoculated with different low pathogenic avian influenza viruses (LPAIV) and highly pathogenic avian influenza viruses (HPAIV), including an H4 virus, which is the subtype of the virus in the present study. The added value of the present work is the transmission experiment between mallards and starlings, which show that starlings can get infected via contaminated water. However, information about how the experimental set-up was designed to prevent cross-contamination between the different groups is lacking (please see below for more detailed questions about that specific point) and it makes it difficult to assess how reliable the results of this experiment are.

**--Significant effort was taken to avoid cross-contamination. We detail our methods and edits in Part II below.**

the authors conclude quite firmly that “starlings have the potential to act as IAV bridge hosts”, while only one “side” of the bridge was studied in this study (the mallard to starling transmission but not the starling to poultry transmission). Given the fact that there was no contact transmission observed between starlings and very limited cloacal/fecal shedding, it is very well possible that transmission from starling to poultry would also be inefficient, making starlings dead end hosts rather than bridge hosts.

**We added additional verbiage to emphasize that our work only shows that starlings have the potential to act as bridge hosts by adding a “may” and with additional text suggesting that the logical next step is to gather evidence about whether infected starlings might infect poultry.**

**Updates:**

**--In summary, we demonstrated that starlings may have the potential to act as IAV bridge hosts if they share water with IAV-infected waterfowl. However, starlings are unlikely to act as maintenance hosts due to limited, if any, intraspecies transmission. In addition, starlings have a relatively brief antibody response which should be considered when interpreting serology from field samples. Further study is needed to evaluate the potential for transmission from starlings to poultry**

**--** **Thus, starlings may have the potential to act as IAV bridge hosts but are unlikely maintenance hosts.**

**-- Overall, these results indicate that while starlings are unlikely to act as maintenance or reservoir hosts for IAVs due to limited intraspecies transmission, this species has may have the potential to act as a bridge host if exposed to IAVs in natural settings.**

**--** **Future evaluation of transmission from IAV-infected starlings sharing resources with poultry is a clear next step to evaluate would provide additional evidence in the assessment of starlings as likely bridge hosts for IAVs between waterfowl and poultry. Further, if transmission to poultry is confirmed, starlings will warrant further scrutiny to identify factors that may impact IAV dynamics such as other virus strains, sex, age, and immune status.**

I think that this point should be discussed more in depth in the manuscript to balance the conclusions of the study.

**We updated the text to explicitly acknowledge the potential for inefficient transmission to poultry:**

**Inoculated starlings did not readily transmit the virus among conspecifics which may indicate potentially inefficient transmission to poultry.**

**Reviewer #2**: The paper by Ellis and colleagues reports on a series of experiments designed to shed light on the role of European starlings in influenza a virus transmission at the wildlife -domestic bird interface. The authors use a local low pathogenic influenza virus (LPIAV) in three experiments that investigate susceptibility to infection and shedding, infection by IAV contaminated water and intraspecies transmission in the species. In addition, one experimental group was employed to test for antibody persistence over time. The experimental methods are sound, and experiments follow a logical chronologic order and tackle key basic questions with view to the potentials of the species as bridge or reservoir host for avian influenza virus. The study is outlined clearly, and results are informative and discussed in some detail.

I have a few concerns and questions regarding the study design, and interpretation of the results.

1- Could the decision to use an LP H4N6, as performing similar experiments in a BSL-3 facility is much more costly and complicated, and as it may be close in dynamics to field situations to H5/H7LPIAV have influenced some of the study results, such as antibody titres and `persistence and shedding?

--**We agree that the choice of virus can have a significant impact on infection dynamics. Nonetheless, while we don’t have comparative data for LP H5s or H7s in starlings, experimental infection studies of mallards with LP H5 and H7 viruses show dynamics similar to those seen for H4 viruses (e.g., see Jourdain etal (2010), VanDalen etal (2010), and Achenbach etal (2011). Moreover, the significant heterogeneity of infection dynamics within subtypes ranging from non-infectious to highly pathogenic (e.g., Gillim-Ross L, Santos C, Chen Z, Aspelund A, Yang CF, Ye D, Jin H, Kemble G, Subbarao K. Avian influenza H6 viruses productively infect and cause illness in mice and ferrets. J Virol. 2008 Nov;82(21):10854-63. doi: 10.1128/JVI.01206-08. Epub 2008 Aug 20. PMID: 18715930; PMCID: PMC2573192) indicate no single virus strain is representative of a subtype.**

2- Why have the authors used detection of viral RNA, expressed as EID50 equivalents/ml as proxy for shedding? With view to the potential of starlings as bridge hosts it would be more interesting to obtain the amount of infectious virus shed by the birds via different routes. Shedding of RNA does not necessarily imply that infectious virus is shed. This could also explain the lack of intraspecific transmission in the third experiment.

--**We agree that detection of viral RNA does not provide equivalent information to measuring infectious virus and that measures of infectious virus add value. We also agree that we should acknowledge the potential for non-infectious virus to have played a role in the lack of contact transmission and have updated the manuscript accordingly. However, we believe molecular detection is an appropriate technique for the current study for multiple reasons: 1) molecular detection has become an accepted standard as a proxy for infection dynamics in experimental studies of wildlife, 2) multiple studies show that qPCR results are generally significantly correlated with infectious titers, 3) the main focus of the current study is transmission and not quantitative dynamics, and 4) we collected nearly 2000 samples for this study so a high throughput method was preferred.**

**--Updated verbiage:**

**Of note, we confirmed concentrations based on detection of viral RNA with RT-qPCR, but infectious virus concentrations may have been lower and may have played a role in the lack of contact intra-species contact transmission.**

3- Were all starlings used in the experiments caught at the same time, and if so, at which time of the year? From the pictures (plumage) it appears that no juveniles (hatch-year) were among the birds used in the experiment. Would the authors expect a different outcome with juveniles?

**--The starlings used in the experiments were captured over a brief period in the fall and our experiments commenced in May after quarantine, acclimation, and pre-screening. We have assessed the impact of age on infection dynamics in mallards and found that age did matter with naïve juveniles shedding less compared to naïve reproductively mature birds (3 and 6 mos of age, see the VanDalen etal study referenced above). Costa etal (2010) also found age could affect viral shedding. While we are not aware of any data available on the impact of age on IAV infections in starlings, we assume that reproductively immature birds might shed less compared with adults, similar to mallards. However, unlike mallards in the wild, because starlings have been shown to be exposed to IAVs less frequently, we would not expect the same differential in exposure status for HY versus AHY individuals as is seen in mallards and other waterfowl and which leads to a relatively higher number of detections in juveniles compared to adults.**

**--In order to generally address this comment and other factors that we did not control for in the study, we added the following verbiage to the ms:**

**Future evaluation of transmission from IAV-infected starlings sharing resources with poultry is a clear next step to evaluate starlings as likely bridge hosts for IAVs between waterfowl and poultry. Further, if transmission to poultry is confirmed, starlings will warrant further scrutiny to identify factors that may impact IAV dynamics such as other virus strains, sex, age, and immune status.**

________________________________________

Part II – Major Issues: Key Experiments Required for Acceptance

**Reviewer #1**: - What was the procedure to ensure that there was no cross-contamination of the naïve starlings in the mallard/starling and starling/starling transmission experiments by the researcher themselves walking from one pen to another?

**--We took extensive measures to prevent cross contamination. We always sampled all starlings before entering the infected mallard pen and we entered the starling pens in the same order each day. We wore rubber boots and had disinfectant footbaths outside each pen which we used upon exit of each pen and prior to entry of a subsequent pen. We also changed gloves and disinfected our sleeves and plastic aprons between pens. and disinfected our gloves between sampling individuals within a pen. We did not share equipment between pens (e.g., nets for capture, boxes for fecal samples). We also limited entry and egress into the pens to limit traffic in the animal room. Besides our efforts to prevent cross-contamination, the lack of starling-starling contact transmission indicates cross-contamination between starling pens was unlikely.**

**--While we did not add extensive technical details to the manuscript, we did add the following verbiage to the methods section:**

**In this and subsequent experiments, we took significant measures (e.g., foot baths, changing PPE, limited entry and egress) to prevent cross-contamination between pens within the transmission cage.**

Were researchers exchanging PPE (including shoes or cover shoes to prevent contamination through feces) in between the groups?

**--As described above we only exchanged gloves between pens, but we did disinfect our boots, sleeves and aprons. Unlike mallards, starlings have very dry feces and we cleaned the feces out frequently to avoid build-up. The only significant viral detection was in oral swabs so that was the more likely source of any contamination.**

Were the naïve birds present during the inoculation of the donor animals or were they placed some time after the inoculation to prevent cross-contamination at the moment of the inoculation?

**--The mallards were inoculated prior to transferring starlings to the room. We inoculated using a pipet and capped the virus between inoculations to limit aerosols. As there were only three mallards per replicate the inoculations only took a few minutes and there was limited opportunity for the virus to aerosolize.**

- All virus titers were based on extrapolation of RT-qPCR results to EID titers based on a standard curve performed with another virus (an H1N1 avian influenza virus). This approach has unfortunately several limitations. First, if this approach is chosen, I think that the same virus should be chosen to perform the standard curve as DI formation can vary between viruses.

**-qPCR detects a highly conserved portion of the matrix gene such that the subtype of the virus should not significantly influence detection. We recently informally compared calibration curves for 2 stock viruses (H3N2 and H1N1) and 4 wild bird viruses (the H4N6 used here as well as an H1N1, H3N8, and H6N2) and the curves were nearly identical across the viruses despite differential passage histories. We report our calibration in terms of EID equivalents because we think it is more interpretable with respect to transmission compared to copy number which is the other common approach to reporting qPCR data. Moreover, many avian IAV researchers merely report Ct-values which are not calibrated or linearized so we believe our approach is more informative. Finally, as previously mentioned, qPCR data generally correlate with infectious titers so we believe the qPCR data are informative for this first comprehensive evaluation of IAV in starlings.**

Second, the different numbers of DIs in the virus stock used as a standard and the swab samples, the detection of RNA from non-infectious viruses and dead infected cells will skew the standard curve. I think that this study would benefit from the determination of actual EID titers by titrating at least some samples in eggs.

**--We agree that the level of DIs can affect quantification; however, infectious titers can also be skewed by the proportion of DIs. Research on DIs for IAVs have shown that DIs can play an important role in infections through complementation where a single cell is infected by two DIs and infectious particles are produced. Thus, plaque assays and the like can underestimate the infectiousness of a viral stock when there is no opportunity for complementarity in infection. In either case, we believe most researchers understand the limitations and trade-offs of molecular detection and the general dynamics reported in our ms are informative even if qPCR results are not an exact quantification of infectious virus.**

- The choice of the virus was not motivated based on what subtype would be the most relevant to study, but on ease to work with lower containment viruses. This comment probably stems from my lack of knowledge on the biocontainment regulation in the US, but is it the case that LPAIV H5 and H7 have to be handled at different biocontainment than LPAIV H4 viruses? Otherwise, studying a LPAIV virus that is more relevant for poultry (H5, H7 or. H6) would have been more interesting.

**--In the US LP H5 and H7 viruses require BSL3 containment for animal studies involving viral replication. As noted in our previous response, we believe intra-subtype heterogeneity is significant such that the general dynamics for an H4 virus are likely to overlap with at least some LP H5, H6, or H7 viruses. Obviously, HP viral dynamics would differ but since we are interested in potential transmission from waterfowl to poultry, LP viruses are important. Moreover, we have significant data on H4 and H6 infections in mallards and for that species, infection curves are very similar for wild bird viruses.**

**Reviewer #2**: Not applicable

________________________________________

Part III – Minor Issues: Editorial and Data Presentation Modifications

**Reviewer #1**: - It is not clear from the description in the material and methods at what biocontainment level were the experiments performed. In the picture in figure 3, the water transmission set-up appears to be placed outdoor. However, in figure 4, the set-up is indoor.

**--The experiment was conducted in BSL2 containment. Thank you to the reviewer for pointing out the oversight. We have updated the methods as follows:**

**During infection testing, all birds were housed in a Biosafety Level 2 (BSL-2) animal room equipped with a four-quadrant transmission cage, custom designed for experimental studies of pathogen transmission (Fig 1).**

**We also added the following text to the Fig 1 caption for clarity:**

**Note: all experimental infections were conducted in a Biosafety Level 2 animal room and the full transmission cage is only shown outdoors for perspective.**

- How long before the start of the experiment were the birds tested for sero-negativity? How long were the starling kept outdoor in between the time they were catch from the wild and the experiment?

**--The starlings were tested multiple times to ensure they were seronegative, including several days before the experiment. The birds were held outdoors for approximately 6 months. The birds were not housed in an open flight pen but within outdoor cages contained within an outdoor building (i.e., a building with no solid walls) with little to no opportunity for contact with other birds. Further, animal care technicians wear PPE upon entry of outdoor buildings and there are footbaths at all entry ways. Nonetheless, the best evidence that the birds were not previously exposed is the serological data. We generally test wild-caught birds on d4 to catch any potential prior infections. If a bird has been previously exposed we would expect to see an amnestic response with a rapid jump in antibody levels. However, all birds were negative on d4.**

- “While there is no evidence….similar to low pathogenic H5/H7 strains”. A reference should be added here to support this statement.

**--We have added the following citations to support this statement:**

**While there is no evidence that H4 IAVs cause a significant threat to wildlife, poultry, or human health, the infection kinetics of these viruses are generally similar to low pathogenic H5/H7 strains (e.g., compare infection dynamics in [23] and [24]).**

- Results: “The primary site of viral RNA excretion was the oral cavity with oral swabs showing significantly higher levels…”. No statistical analysis was conducted to support this statement.

**--To avoid the suggestion that a statistical test was performed, we replaced the word “significantly” with “considerably.”**

- Figure 1: the variation across individuals for the fecal and cloacal shedding should be also depicted.

**--We agree that it is ideal to show indicators of variability in figures; however, we did not update the figure to show individual variability in fecal and cloacal shedding. We tried many versions of the figure that included the variability but could not find a solution that led to a readable figure. We considered breaking the figure into 3 independent plot so that we could visualize variability around each shedding curve, but when we did so the comparison of the mean shedding for the three routes was less obvious and we decided that was a higher priority given that the data will be publicly available for anyone interested.**

- Inoculation of the starlings: it would be useful to understand why it was chosen to inoculated the starling via the eyes, in addition to the choanal route. After 4 hours, was only the eye inoculation repeated in the other eye, or was the choanal inoculation also repeated?

**--We inoculated both orally and intra-ocularly as that is a common approach used in IAV inoculation of poultry and other species where shedding is not primarily from the GI tract. We inoculated both the other eye and oral-choanally after 4 hours. We have updated the text to state this more explicitly:**

**Specifically, we delivered a single drop from a pipet to one eye and the remainder oro-choanally. We repeated the procedure approximately four hours later, applying the inoculum to the other eye as well as oro-choanally.**

- Figure 5 and 6: data from the different groups should not be combined in one box plot graph.

**--As suggested, we updated the figures to remove the boxplots illustrating mean behavior across the replicates for both Figure 5 and Figure 6.**

- Author summary: “highly pathogenic to poultry, wild birds and humans. In fact, the last three major pandemics were caused by IAVs with genes derived by bird viruses”. It seems that here the authors refer to two different kind of evolution of avian influenza viruses, but yet link in these two sentences as if they were related, which is not the case. The first one is the transition from low to highly pathogenic avian influenza in poultry. These viruses are highly pathogenic in poultry but not necessarily in wild birds or humans. The second is reassortment between two influenza A viruses.

**--We agree with the reviewer that the two ideas were conflated in the text. The issue of avian IAV genes contributing to pandemics speaks to the general importance of studying avian viruses, but is tangential to the point that it is important to understand how wild bird viruses are introduced to poultry. Therefore we have deleted the second sentence from the author summary.**

- Introduction: in the sentence where ref 19 is cited, it would be good to indicate the subtype of the influenza virus that was detected in the starling.

**--We updated the ms to specify that the subtype was H5.**

- Figure 2 could be split in different panels to increase readability.

**--We produced a number of plots to show the data as a combined plot or as multiple panels and then polled a number of people about readability and more people preferred the combined plot so we have left the data as they are. One issue when we presented the data in panels is that the data were collected across very different timeframes so when they were separated we either had to stretch the data out so they did not line up or use a lot of white space. We also considered only showing the long-term data in the plot, but most folks preferred showing all the data.**

**Reviewer #2**: Introduction

Page 10: Please provide a reference for this assumption “While there is no evidence that H4 IAVs cause a significant threat to wildlife, poultry, or human health, the infection kinetics of these viruses are generally similar to low pathogenic H5/H7strains.”

**--We have added the following citations to support this statement:**

**While there is no evidence that H4 IAVs cause a significant threat to wildlife, poultry, or human health, the infection kinetics of these viruses are generally similar to low pathogenic H5/H7 strains (e.g., compare infection dynamics in [23] and [24]).**

**Citations**

Jourdain E, Gunnarsson G, Wahlgren J, Latorre-Margalef N, Bröjer C, Sahlin S, et al. (2010) Influenza Virus in a Natural Host, the Mallard: Experimental Infection Data. PLoS ONE 5(1): e8935 <https://doi.org/10.1371/journal.pone.0008935>

VanDalen KK, Franklin AB, Mooers NL, Sullivan HJ, Shriner SA (2010) Shedding Light on Avian Influenza H4N6 Infection in Mallards: Modes of Transmission and Implications for Surveillance. PLoS ONE 5(9): e12851 <https://doi.org/10.1371/journal.pone.0012851>

Achenbach JE, Bowen RA (2011) Transmission of Avian Influenza A Viruses among Species in an Artificial Barnyard. PLoS ONE 6(3): e17643

Costa TP, Brown JD, Howerth EW, Stallknecht DE. The effect of age on avian influenza viral shedding in mallards (Anas platyrhynchos). Avian Dis. 2010 Mar;54(1 Suppl):581-5. doi: 10.1637/8692-031309.
